# Porous Hydrophobic–Hydrophilic Composite Hollow Fiber and Flat Membranes Prepared by Plasma Polymerization for Direct Contact Membrane Distillation

**DOI:** 10.3390/membranes11020120

**Published:** 2021-02-08

**Authors:** Ashok K. Sharma, Adam Juelfs, Connor Colling, Saket Sharma, Stephen P. Conover, Aishwarya A. Puranik, John Chau, Lydia Rodrigues, Kamalesh K. Sirkar

**Affiliations:** 1 Applied Membrane Technology Inc., 11558 Encore Circle, Minnetonka, MN 55343, USA; aksharma@appliedmembranetech.com (A.K.S.); juelf002@umn.edu (A.J.); ccolling@appliedmembranetech.com (C.C.); saketsharma@appliedmembranetech.com (S.S.); 2 Otto York Department of Chemical and Materials Engineering, New Jersey Institute of Technology, University Heights, Newark, NJ 07102, USA; aap253@njit.edu (A.A.P.); jc56@njit.edu (J.C.); lr59@njit.edu (L.R.)

**Keywords:** membrane distillation, composite membrane, plasma-polymerized hydrophobic fluorosiloxane coating, hydrophilic porous hollow-fiber substrate

## Abstract

High water vapor flux at low brine temperatures without surface fouling is needed in membrane distillation-based desalination. Brine crossflow over surface-modified hydrophobic hollow fiber membranes (HFMs) yielded fouling-free operation with supersaturated solutions of scaling salts and their precipitates. Surface modification involved an ultrathin porous polyfluorosiloxane or polysiloxane coating deposited on the outside of porous polypropylene (PP) HFMs by plasma polymerization. The outside of hydrophilic MicroPES HFMs of polyethersulfone was also coated by an ultrathin coating of porous plasma-polymerized polyfluorosiloxane or polysiloxane rendering the surface hydrophobic. Direct contact membrane distillation-based desalination performances of these HFMs were determined and compared with porous PP-based HFMs. Salt concentrations of 1, 10, and 20 wt% were used. Leak rates were determined at low pressures. Surface and cross-sections of two kinds of coated HFMs were investigated by scanning electron microscopy. The HFMs based on water-wetted MicroPES substrate offered a very thin gas gap in the hydrophobic surface coating yielding a high flux of 26.4–27.6 kg/m^2^-h with 1 wt% feed brine at 70 °C. The fluxes of HFMs on porous PP substrates having a long vapor diffusion path were significantly lower. Coated HFM performances have been compared with flat hydrophilic membranes of polyvinylidene fluoride having a similar plasma-polymerized hydrophobic polyfluorosiloxane coating.

## 1. Introduction

Water evaporation through a porous hydrophobic membrane is being intensively investigated for water desalination and a few other water treatment processes. This process, termed membrane distillation (MD), has several variants depending on how water evaporated from hot brine on the feed side of the membrane is condensed on the other side of the membrane. We are here concerned primarily with the direct contact membrane distillation (DCMD) process, where the evaporated water is condensed by a flowing stream of cold distilled water on the other side of the membrane. There are excellent and comprehensive review articles on various aspects of MD and DCMD [1,2,3,4,5,6,7], the last one being the earliest containing a mini-review of MD. There may be occasional literature references here to other MD varieties: vacuum membrane distillation (VMD), air gap membrane distillation (AGMD), and sweep gas membrane distillation (SGMD).

In DCMD, a porous gas-filled hydrophobic membrane exists between hot brine and cold distillate streams. Water evaporated at the hot brine-membrane interface is transported through gas-filled pores to the other side where it condenses in the flowing cold distillate. Water vapor flux depends on the physical properties of the porous membrane, the temperatures of the two water streams, extent of temperature polarization in the two flowing liquids, the rate of conductive heat loss as well as any membrane surface fouling. Additional issues of importance involve the extent of water recovery. A few major recent developments in these and other aspects of MD will be identified first before we converge on the subject of this work involving water vapor flux in surface-modified hollow fiber membranes.

Water flux in MD depends on the water vapor pressures and therefore the temperatures of water in contact with the two sides of the membrane. Conventionally, in MD, the hydrophobic membrane is contacted with hot brine from an external source to generate water vapor at the water-membrane interface. Such a process does lead to temperature polarization wherein the bulk brine temperature is higher than the temperature at the brine-membrane interface, which leads to a lower water vapor pressure-based driving force. To enhance MD flux, Chen et al. [8] pursued breaking hydrogen bonds in water via gold nanoparticles-adsorbed ceramic rods (AuNPs@CRs) and enhancing the water vapor pressure. Techniques that use other sources to heat the membrane-water interface region e.g., “self-heating” membrane by a high-frequency magnetic field [9], electrically conducting MD system heated by Ohmic resistance at alternating currents [10], avoid the need for preheating bulk feed brine and, therefore, claim lower intrinsic heat consumption. These techniques also do not suffer from negative consequences of temperature polarization since membrane surface temperature is higher than bulk brine temperature (on the other hand, there is heat loss to the bulk water). However, such claims are not relevant for cases where DCMD is employed to utilize waste heat already available in a hot brine (e.g., produced water [11]) or where the feed brine is heated via a solar collector. Enhancement of water vapor flux and stable operation then become more important.

A major problem in DCMD is membrane fouling, which can lead to reduction in salt rejection as well as reduced flux. Membrane pores may undergo wetting and, therefore, the process may need to be shut down and the membrane regenerated. Membrane fouling may be due to a variety of phenomena: scaling salt fouling, silica fouling, organic fouling, particle fouling, colloidal fouling, or biofouling. Particular sources of fouling may be mitigated under certain conditions as shown in the following studies.

To prevent fouling by scaling salt precipitates, the following technique using hollow fiber membranes (HFMs) appears to have been successful [12,13,14]. Hot feed brine was directed in cross flow across the outside surface of porous hydrophobic polypropylene (PP) HFMs in rectangular modules. The HFMs had a thin highly porous hydrophobic plasma-polymerized fluorosiloxane coating on the outer surface. In laboratory studies as well as pilot plant operation to concentrate seawater, the near-superhydrophobic membrane surface (contact angle ~140° [13]) was not fouled; although precipitates of scaling salts were floating all around, water flux was unaffected [12,13,14]. HFM-based cross flow modules specifically designed to have hot brine flow over and around HFMs create numerous flow separation points, which in turn generate secondary flows, which spontaneously scrub the membrane surface and prevent membrane fouling. In treatment of produced water containing a variety of salts as well as silica, 80% of water was recovered; no silica fouling of the membrane was observed [11].

Water-soluble agents, such as alcohols and surfactants, which reduce the surface tension of the solution, can easily wet the pores of most hydrophobic membranes in DCMD; surfactants take time to wet the pores but alcohols do it instantly [15]. Porous polyvinylidene fluoride (PVDF) membrane surfaces were modified by CF_4_ plasma treatment to make superhydrophobic membrane surface [16], which exhibited negative charges on the surface, as well as stable MD performance in concentrating anionic surfactant-emulsified waste water; however, the membrane suffered from severe wetting when cationic surfactant was used. Stick water waste from meat rendering operations (rich in proteins, fats, and minerals) end up wetting hydrophobic membrane pores due to the fats. The fats got attached to membrane pores and facilitated liquid flow through the hydrophobic polytetrafluoroethylene (PTFE) membrane, which showed a total loss of flux within 0.5 h [17].

However, commercial hydrophobic PTFE membranes with a hydrophilic polyurethane (PU) surface layer were used successfully in DCMD on real poultry, fish, and bovine stick waters [17]; a metal microfiltration membrane was used to capture fats prior to DCMD. Tang et al. [18] showed how hydroxide ion generation, driven by water electrolysis on the electrically conducting membrane surface consisting of carbon nanotubes and a polyvinyl alcohol layer over a hydrophobic layer, can be used to efficiently dissolve silicate scaling that developed during the process of desalinating the geothermal brine by MD, negating the need for chemical cleaning. Note: a previous study employing coated HFMs with porous polyfluorosiloxane coatings operated with crossflow mode of hot produced water containing silica did not show any fouling [11].

It is known that in one pass of the brine through a DCMD unit, one cannot evaporate more than a limited fraction of the feed water. He et al. [19] pointed out that one can evaporate anywhere between 7 and 9% of the feed water in one pass, depending on the thermal efficiency of the DCMD unit for hot incoming brine at 90 °C and exiting at 35 °C. This has also been emphasized by Winter et al. [20]. Therefore He et al. [19] developed a concentration cascade of DCMD units with heat exchangers to achieve water recovery upwards of 60%. Such a cascade involves integration of multiple DCMD units with multiple heat exchangers to recycle the heat.

In AGMD, one usually achieves a higher thermal efficiency than DCMD due to the air gap, which also reduces the flux. By using an AGMD module having two adjoining and well-mixed sets of hollow fibers—one porous and hydrophobic for MD, and the other being a solid hollow fiber with a water-impervious wall to allow a coolant to flow through its fiber bore as it condenses the distillate vapor in the intervening shell-side space—a much higher module production rate can be achieved [21] than that in other AGMD designs. However, fractional water evaporation achieved in one pass is still limited. A flat plate-based new configuration called Feed Gap Air Gap MD (FGAGMD) has been designed [22] in which the feed brine is separated from the heating stream by a heat exchange surface; the cooling liquid is also separated from the permeate channel as in AGMD. Such a channel arrangement has allowed achievement of high one pass water recovery varying between 32–93% depending on the feed and its salt concentration.

All techniques in DCMD rely so far on a porous hydrophobic membrane layer to evaporate water on one surface and condense water at the other surface. The thinner this membrane layer is, the higher the flux [23]; a thinner membrane layer leads to a higher conductive flux loss, which depends however on the temperature difference between the two sides. With a very thin hydrophobic layer and a low ∆T between the two surfaces, conductive heat loss can be minimized, and the thermal efficiency can be high in DCMD (as much as 86%); yet the flux can still be reasonable [24]. Such a thin porous hydrophobic layer may be realized in a number of ways, e.g., plasma polymerization, polymer coating/grafting, and using hydrophobic fluorinated surface modifying macromolecules (SMMs) in the casting solvent used to fabricate a porous hydrophilic membrane [25,26,27,28].

A thin layer of polyvinyl alcohol (PVA) was coated successfully on a porous PTFE membrane by solution dipping followed by a cross-linking step to reduce the gas-filled pore length by a hydrophilic coating of polyvinyl alcohol. The water flux achieved with a 30 g/L salt solution was 12.2 L/m^2^-h with a salt rejection of 99.9% for a feed temperature of 60 °C and distillate temperature of 17 °C [29]. A novel dual-layer flat membrane consisting of a thin hydrophobic top-layer of PVDF and a relatively thick hydrophilic PVDF-PVA sub-layer was fabricated using the non-solvent thermally induced phase separation (NTIPS) method [30]; the water vapor flux achieved was quite high.

A porous hydrophilic membrane prepared by casting from a NMP solution of polysulfone blended with polyvinyl pyrrolidone was converted into a hydrophobic membrane by treating it with CF_4_ plasma [31]. The gaseous plasma modified not only the exposed membrane surface making it hydrophobic, but also the interior of the membrane pores; this was confirmed by the SEM–energy-dispersive X-ray spectroscopy (EDX) data of the cross section. Using commercially available flat polyethersulfone (PES) membrane (MicroPES 2F), Eykens et al. [32] explored a variety of coating techniques including vacuum plasma technology and atmospheric plasma technique to develop efficient hydrophobic–hydrophilic structures for successful DCMD. The CF_4_ plasma treatment technique to develop a hydrophobic layer on existing asymmetric hydrophilic polyethersulfone membranes in both flat and hollow fiber forms was implemented by Wei et al. [33] for DCMD studies. Interestingly, this process ended up completely hydrophobizing the substrate including the back surface.

A major factor in such plasma treatment involves the extent of plasma exposure. Puranik et al. [34] studied the performance of flat hydrophilic PVDF membranes exposed to vacuum-based plasma polymerization depositing a polyfluorosiloxane coating as a function of treatment duration; by design, the plasma treatment duration was limited. The DCMD fluxes achieved were high. Here we report the DCMD performances of porous hydrophilic MicroPES hollow fibers of PES coated on the outside diameter (OD) with a plasma polymerized hydrophobic polyfluorosiloxane or polysiloxane coating. The performances of such coated hollow fiber membranes (HFMs) have been compared with hydrophobic PP based hollow fibers having a similar plasma polymerized coating. The gas gap in such PP-based hollow fibers through which water vapor diffuses is, however, very large compared to the very thin gas gap in the MicroPES hollow fibers with a plasma-polymerized coating, since the MicroPES hollow fiber is spontaneously wetted with water. Detailed performance results of a few flat hydrophilic porous PVDF membranes with a porous hydrophobic plasma polymerized coating of polyfluorosiloxane (not reported in [34]) are also provided here for comparison.

## 2. Experimental Materials and Methods

### 2.1. Materials and Chemicals

Hydrophilic PES hollow fibers of MicroPES type obtained from Membrana (Charlotte, NC, USA) were coated with an ultrathin plasma polymerized layer of hydrophobic polyfluorosiloxane or polysiloxane polymer on the outside surface. These fibers were asymmetric in structure with finer pores on the OD where plasma modification was done. Porous hydrophobic PP hollow fibers (PP 150/330) were also acquired from Membrana (Charlotte, NC, USA) and coated on the OD with an ultrathin plasma polymerized layer of hydrophobic polysiloxane or polyfluorosiloxane polymer using a continuous plasma process operated at 13.56 MHz. The monomer feed consisted of siloxane or blend of siloxane and perfluorohydrocarbon monomers selected from a group of 1,1,3,3 tetramethyldisiloxane (TMDSO), 1,3,5,7 tetramethylcyclotetrasiloxane (TMCTS), hexafluoroethane (HFE), perfluorohexane (PFHX), or perfluorooctane (PFOC). The monomer flow rate varied from 10–40 SCCM, the residence time in the reactor varied from 15 to 30 s and system pressure from 20–100 mTorr. The discharge power varied from 10–150 W. The basic properties of the uncoated porous hollow fiber membranes and their dimensions are provided in Table 1. Porous hydrophilic flat membranes of PVDF were obtained from MilliporeSigma (Bedford, MA, USA) and Pall Corp (Port Washington, NY, USA). The relevant properties of these two membranes are also listed in Table 1. These flat membranes were used as substrates for plasma coating. A highly porous ultrathin hydrophobic coating of polyfluorosiloxane polymer was deposited on these membranes by vacuum-based plasma polymerization [34].

Puranik et al. [34] have provided details of the plasma exposure variation between different samples of flat PVDF substrate membranes. The fluorosiloxane polymers on flat films were deposited in a tubular batch reactor under reaction conditions similar to those used for the HFMs, except that the reaction times were slightly longer. The samples AKS-6591-1M, AKS-6591-2M underwent plasma exposure for 1 min at two different positions in the reactor. The suffix -1 refers to a pre-electrode reactor position and the suffix -2 refers to the post-electrode reactor position. It is expected that position -2 would produce more cross-linked polymer than reactor position -1. Samples AKS-6592-1M, AKS-6593-1M, AKS-6594-1M were prepared by treating substrates for 2, 3, and 6 min respectively with plasma in the pre-electrode region.

### 2.2. Hollow Fiber Membrane Mini-Modules

A large number of mini-modules were built using both PP and PES hollow fiber membranes whose surfaces were modified by plasma polymerization. Each module contained 20 HFMs; each HFM was ~18 cm long. The total membrane surface area for each PP hollow fiber-based module was 7.12 × 10^−3^ m^2^ whereas that for each PES hollow fiber-based module was 5.65 × 10^−3^ m^2^ (area is based on the OD of the HFMs). The cylindrical shell of each module had an internal diameter (ID) of 0.95 cm and was made of polycarbonate. The HFM modules were regular parallel flow without rectangular crossflow [12,13,14] or radial crossflow arrangements [35] used in commercial scale modules to facilitate fouling-resistance and higher transfer coefficients/fluxes. The empty module shell volume was ~15.32 cm^3^. The HFM packing density for PP HFs in the module was 8.78% and that for MicroPES HFs was 5.17%.

### 2.3. Methods and Procedures

**Figure 1:**
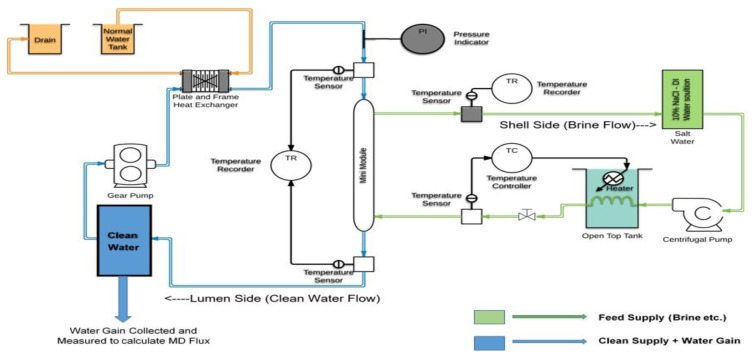
Experimental direct contact membrane distillation (DCMD) set up for studying the desalination performances of plasma coated hollow fiber membrane mini-modules.

The schematic for the experimental set up for studying the DCMD performances of plasma-coated hollow fiber membrane mini-modules is shown in Figure 1. It has two parallel flow loops: one for the brine to flow on the shell side of the membranes and the second loop for the distillate to flow through the lumen of the same membranes. In the membrane module, the brine stream and the distillate stream flow counter-currently. All flow loop components (e.g., instruments, pumps, sensors, tubing, valves, etc.) are made of materials that do not rust or contaminate the system.

Heated feed brine was stored in a polypropylene tank; its temperature was maintained by using a heat/temperature controller. It was pumped out into the shell side of the hollow fiber membrane module and then returned back to the tank. The hot brine inlet and outlet temperatures in the membrane module were measured and recorded using a temperature sensor. In the distillate loop, the distillate tank, filled with a certain minimum quantity of clean distilled water, was kept over an electronic weighing balance. Water from this tank was pumped through a heat exchanger into the module through the hollow fiber lumens counter-currently. The temperatures of the distilled water at the inlet and the outlet of the module were also measured using temperature sensors.

The distillate stream exiting the module was returned to the clean water tank. Any weight change in the tank was continually monitored and recorded. Simultaneously, the salinity/conductivity of the distillate was recorded continually using a probe. The feed NaCl concentrations were: 1 wt%, 10 wt%, and 20 wt%; feed brine temperature was varied between 43.3 °C (110 °F) to 70 °C (158 °F). The incoming distillate side cold water temperature varied from 22.8 °C to 29.4 °C (73 °F to 85 °F) depending upon the heat exchanger, room temperature and, more importantly, the inlet brine temperature and the membrane distillation rate.

The DCMD experimental setup for flat membranes has been described in references [11,12,23,34]. A chlorinated polyvinyl chlroide (CPVC) based rectangular cell described in [23] with a membrane area of 0.0011 m^2^ was used for the flat membranes. Studies with flat membranes used 1% NaCl solution. In most experiments, the incoming hot brine temperature varied between 60 °C and 85 °C. The distillate-in temperature was generally around 20 °C. The distillate production rate was obtained by the overflow rate from the distillate tank by a weighing machine and is obtained from Equation (1):

(1) $$N_{v}\left(\frac{\mathrm{kg}}{m^{2}.h}\right)=\;\frac{Increase\;in\;weight\;of\;water\;\left(=volume\;of\;water\;transferred\left(L\right)*water\;density\left(\frac{\mathrm{kg}}{L}\right)\right)}{membrane\;area\left(m^{2}\right)*time\left(h\right)}$$

To check for any salt leakage to the distillate side, the distillate side conductivity was measured using a conductivity meter (Orion 115A+, Thermo Fisher Scientific, Waltham, MA, USA). Any experiment under given conditions was run for around 3 h after steady state was reached.

Scanning electron microscopy was carried out using JSM 7900F Field Emission SEM (JEOL USA, Peabody, MA, USA) to develop micrographs of the surfaces as well as the cross section of the plasma polymerized coated hollow fiber membrane substrates of both PES and PP. The elements in the plasma-coated region determined via energy-dispersive X-ray spectroscopy (EDX) were reported in earlier studies [34]. Similar Si/F monomers and reaction conditions were used in this series of coatings on HFMs and therefore we expect the composition of these polymers to be similar. Note: due to the unique mechanism of the plasma polymerization process, plasma polymers can have significantly different elemental composition than the monomer used.

For liquid entry pressure (LEP) measurements, distilled water at room temperature was used as essentially stationary feed liquid on the shell side. A specially designed and fabricated LEP Test Apparatus (Applied Membrane Technology Inc., Minnetonka, MN, USA) was used to measure the “Leak Rate” of coated and uncoated membranes at different pressures. No water was present on the permeate side. The pressure settings used for coated PES membranes for leak measurement were 27.7, 48.3, and 68.9 kPag (4, 7 and 10 psig). The pressure of the feed distilled water was held constant for 30 min. If no permeate flow was observed, then the pressure was raised by 20.6 kPa (3 psi) and held constant until the final setting of 68.9 kPag (10 psig) was reached. The pressure at which we observed any permeation of water was considered to be the LEP. The permeation rate was measured at the LEP. For uncoated PES membranes, only 20.6 kPag (3 psig) pressure setting was used due to high leak rate: the hold time could not go beyond 10 min. For coated PP HFM modules, the same pressure settings were used but the hold time ranged from 30 to 60 min at each pressure setting.

## 3. Results

A total of 61 PP mini-modules and 24 PES mini-modules were tested for DCMD performance. We report here results of the 6900 series modules built with coated PES hollow fibers. The results from coated PP hollow fiber-based modules with the designations 6700 and 6800 are also being reported. Over 6000 h of DCMD data were collected for both HFM substrates having different porous hydrophobic polyfluorosiloxane/polysiloxane coatings at various feed brine concentrations and temperatures. Feed brine flow rate was ~2.66 L/min; the distillate flow rate was ~ 80 mL/min. Exiting hot brine temperature decreased from that at the inlet by ~0.5–3.3 °C (1–6 °F).

The average DCMD water vapor flux obtained from different PES substrate-based modules are illustrated in Table 2 for feed brine at 70 °C; two brine feed concentrations, 1% and 10%, were used. Of the different modules in this series, modules 6935, 6939 and 6940 yielded high water vapor fluxes between 26.4 and 27.6 kg/m^2^-h with 1% feed brine. Since the feed temperature was 70 °C and these were parallel flow modules, we can reasonably expect significant flux enhancement if we had the hot brine in cross flow. For 10% brine feed at 70 °C, a lower but decent DCMD flux level of 17–19.5 kg/m^2^-h was obtained for this series of coatings at 70 °C. These are high values. We deliberate on these flux values later.

The variable quantity, W/FM, is a composite plasma parameter which determines the average energy used in the plasma process per unit weight of monomer. An increase in the W/FM parameter led to a decrease in distillate flux at both salt concentrations (6935 vs. 6940 and 6933 vs. 6922). It has been demonstrated earlier by Sharma and Yasuda [36,37] that an increase in W/FM changed the kinetics of plasma polymerization and led to an increase in polymer deposition rate or an increase in crosslink density or both. Thus, an increase in W/FM can lead to an increase in the thickness or crosslink density of the coating resulting in a decrease in the distillate flux. Addition of fluoro monomer in the feed made the coating more hydrophobic and even less wettable. It also improved longevity of the membrane although the distillate flux marginally decreased. Halogen based compounds are known to act as catalyst and accelerate the rate of plasma polymer deposition [38]. This is perhaps why the polyfluorosiloxane coating in 6932 led to lower flux compared to the polysiloxane coating in 6935 in spite of similar plasma energy conditions. The fluoro monomer used in 6932 not just copolymerized but also resulted in thicker coating, which further reduced the pore size of the underlying substrate and increased the hydrophobic gap thickness, leading to reduction in the flux. Monomer TMCTS in 6939 also resulted in thicker coating and lower distillate flux due to higher O/Si ratio in the monomer [39].

**Figure 2:**
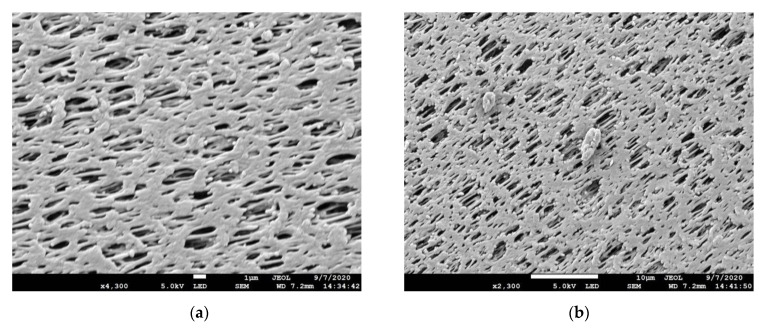

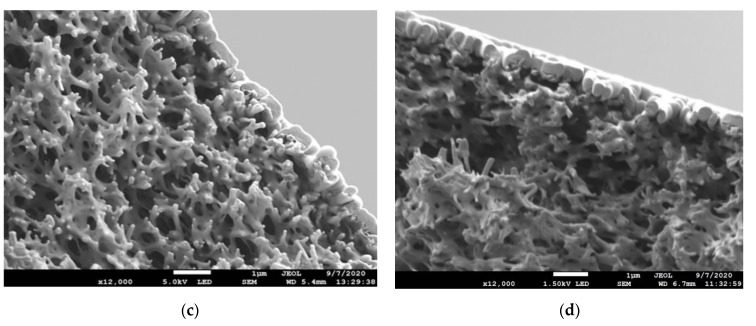
(**a**) SEM picture of the surface of 6939 coating on PES hollow fiber at 4300× magnification. (**b**) SEM picture of the surface of 6940 coating on PES hollow fiber at 2300× magnification. (**c**) SEM picture of the cross section of 6939 coating on PES hollow fiber at 12,000× magnification. (**d**) SEM picture of the cross section of 6940 coating on PES hollow fiber at 12,000× magnification.

Figure 2a–d, shown below, focus on the SEM pictures of the surfaces and cross sections of two candidate hollow fiber membranes in this series with a PES-based HFM substrate. The relevant module numbers from which the hollow fibers were taken out for SEM studies from this series are 6939 and 6940. Figure 2a,c describe respectively the surface and cross section of the 6939 series; the coating on the surface over the porous structure is clearly visible in both figures. Figure 2b,d provide the corresponding views of the 6940 series. From both Figure 2c,d, it is clear that the coating thicknesses are less than 0.4–0.5 µm. The coated pores appear to have a significant size distribution; further, the pores are somewhat elongated and elliptical in nature.

**Figure 3:**
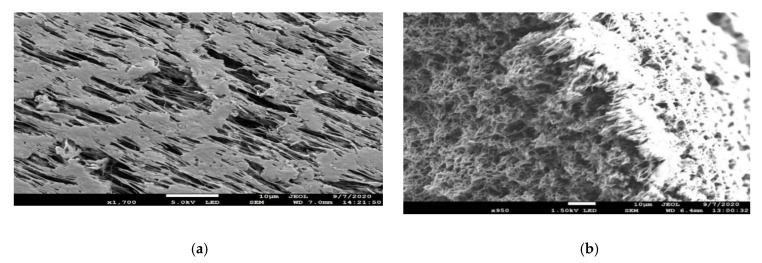
(**a**) Surface of the 6824 coated PP hollow fiber (1700× magnification). (**b**) Cross section of the 6824 coated PP hollow fiber (950× magnification).

Table 3 provides the average water vapor flux values obtained in DCMD with coated PP hollow fiber modules exposed to a 70 °C feed brine at two salt concentrations, 10% and 20%. Two coating series, designated 6700, 6800, were tested. The coating designated 6826 in the 6800 series has the highest flux of 15.5 kg/m^2^-h for a 10 wt% brine feed. The values from other coatings are not too far off. Here again, higher brine concentration (lower vapor pressure) gave lower flux (coating in 6810 vs. 6713) and coating produced at lower W/FM or reduced reaction time offered higher flux in general (coating in 6713 vs. 6712 and coating in 6805 vs. 6804). It has been demonstrated by Sharma et al. [40] that a decrease in reaction time leads to a proportional decrease in polymer coating thickness. The reduced coating thickness is likely to have a lesser decrease in the pore size of the membrane, which would provide higher flux; the gas gap thickness is also reduced. Several different siloxane and fluoro monomers were used for these experiments. It was noted that the polyfluorosiloxane coatings based on PFHX and PFOC, especially the latter, had better film forming properties than HFE due to higher molecular weight and lower F/C ratio and produced thicker coatings and, hence, lower flux. Coating 6826, which was produced by reducing the amount of siloxane monomer in the mix by 50%, resulted in a significant increase in distillate flux. It is because the siloxane monomers, in general, are better film formers than the F-monomers and a reduction in silicone monomer, results in a thinner polymer coating and correspondingly higher flux. Plasma polymer coatings for this series were deposited at relatively lower W/FM because the polypropylene HFM (PP 150/330) could not tolerate high W/FM values especially in presence of F-monomers and became mechanically weak at high-energy conditions. The SEM-based micrographs of the surface and cross section of coating 6824 in the 6800 series are shown in Figure 3a,b, respectively.

The diameters of the coated surface pores in Figure 2a,b vary; a few are somewhat large. As a result, it is expected that the LEPs will be on the low side. Table 4 provides the LEP data and the corresponding leak rates using distilled water for a few types of coated PES hollow fibers. The leak rate for PP hollow fiber is provided for reference only. The LEP value of 48.3 kPag (7 psig) is on the low side. This LEP value is very close to that of flat hydrophilic PVDF substrate (MilliporeSigma; 0.4 µm pore size) having a very light coating, AKS-6591-1M [34]. However, the leak rates are extremely low. By manipulating plasma conditions, the LEP value can be substantially increased as was observed earlier with flat membranes [34]. In Table 5, we provide an idea about the rate of salt leakage as measured by change in conductivity of the distillate at the LEP for PP based hollow fiber membranes. Notice the lower salt leak rate in the polyfluorosiloxane coated PP hollow fiber coating.

In the earlier study using flat hydrophilic PVDF membranes [34], we had observed a very limited flux reduction accompanied by a considerable increase in LEP when the plasma polymerization was just enhanced a bit. For example, flat membrane samples AKS-6591-1M, AKS-6591-2M underwent plasma exposure for 1 min at two different positions in the reactor with more crosslinking at -2 position due to a more intensive plasma exposure; the corresponding LEP values for the two coatings were 62 and 110 kPag (9 and 16 psig) respectively. However, the flux for the membrane modified at -2 position was reduced by only 2 kg/m^2^-h. We expect an almost similar behavior for hollow fiber membranes since the plasma polymerization conditions were very similar. This happens during earlier stages of coating. Figure 6 in reference [34] describes the experimental data for DCMD flux vs. plasma polymerization time.

**Figure 4:**
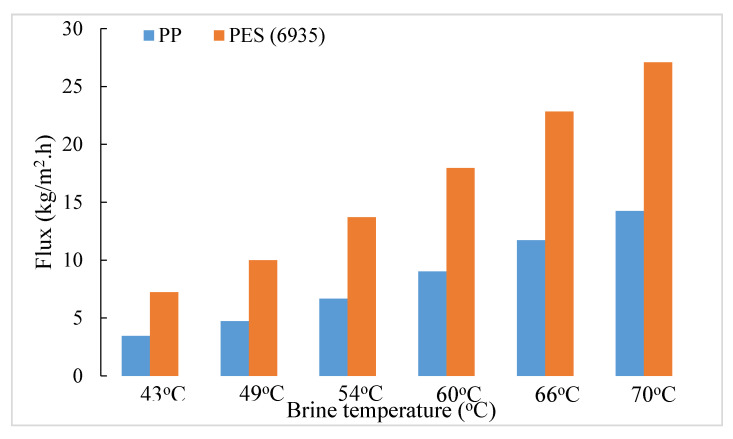
Comparison of average water vapor flux between PP (6713) and PES (6935) based coated hollow fiber modules for 1% brine feed at different temperatures.

**Figure 5:**
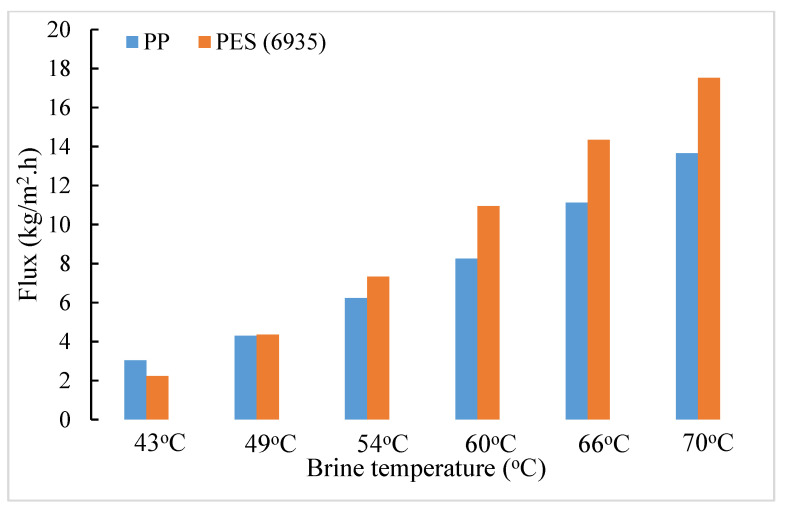
Comparison of average water vapor flux between PP (6713) and PES (6935) based coated hollow fiber modules for 10% brine feed at different temperatures.

A comparison of the flux performances of the two types of hollow fiber substrate is useful since in the hydrophilic PES hollow fibers, the length of the water vapor diffusion path is very small. It essentially spans the length of the porous plasma polymerized hydrophobic coating and tends toward creating conditions for orifice flow. Therefore, the flux level is likely to be higher; however, conductive heat loss is increased, which tends to reduce the flux [23]. In the PP substrate-based coated hollow fibers, the vapor diffusion path is much longer. The fluxes are going to be lower but conductive heat loss will also be significantly lower [23]. The overall performances of the two types of coated HFMs are shown in Figure 4 and Figure 5 for two different salt concentrations, 1 wt% and 10 wt%, respectively.

It appears that the flux levels in coated PES hollow fibers are always higher than those in coated PP hollow fibers for 1 wt% brine feed by a substantial amount. On the other hand, that difference is significantly smaller in the case of a 10% brine feed along with a generally lower level of flux.

Since the HFM packing density for PP HFs in the module was 8.78% and that for MicroPES HFs was 5.17% for a constant module volume, the open volume was significantly larger in the PES HFM modules. The extent of bypassing/channeling of hot brine would be larger in the PES HFM modules. With 1% hot feed brine, the extent of temperature and concentration polarization influenced by this bypassing and therefore water vapor pressure reduction would be much less. On the other hand, the same level of bypassing/channeling would have much larger effect on concentration/temperature polarization with 10% hot feed brine in PES modules and correspondingly larger vapor pressure reduction. Hence, we observe a significantly larger flux reduction with PES modules.

Further, this difference between the two sets of HFs keeps on decreasing as the brine feed temperature decreases with 10% brine feed. There is almost no difference at a feed temperature of 49 °C. The relative flux levels are reversed at a low feed temperature of 43 °C. This is likely due to an enhanced contribution of conductive flux loss vis-à-vis the total heat flux across the membrane (which is the sum of convective heat flux due to vapor transport and the conductive heat flux) for the coated PES hollow fiber membranes. This is aided by more bypassing in the PES modules.

The modules with coated PP hollow fibers, when tested on 1% brine feed, showed very little salt leakage, even after running for more than 1000 h. The distillate conductivity increased only by a few µS/cm. When testing PP hollow fibers on 10% brine feed, the salt leakage increased just a little bit more. Coated HFMs based on MicroPES substrate, particularly 6935, performed well with 1% brine; the very low leakage rate was comparable to that of PP HFMs. For a feed of 10% salt, the leakage was slightly higher, suggesting the need for a longer plasma polymerization time or a higher W/FM to prevent any salt leakage at all.

To provide a comparative perspective on the water vapor fluxes from these coated HFMs vis-à-vis those obtained with flat membranes, we now briefly describe the observed flux behaviors of a few porous flat hydrophilic PVDF membranes having a thin plasma polymerized polyfluorosiloxane coating for 1 wt% salt containing feed. The general characteristics of these membranes in terms of LEP values and overall relative flux behavior vis-à-vis the plasma polymerization time are available in the earlier study [34]. However, detailed flux behavior vis-à-vis feed temperature and flow rates were not available for the specific membranes considered here.

**Figure 6:**
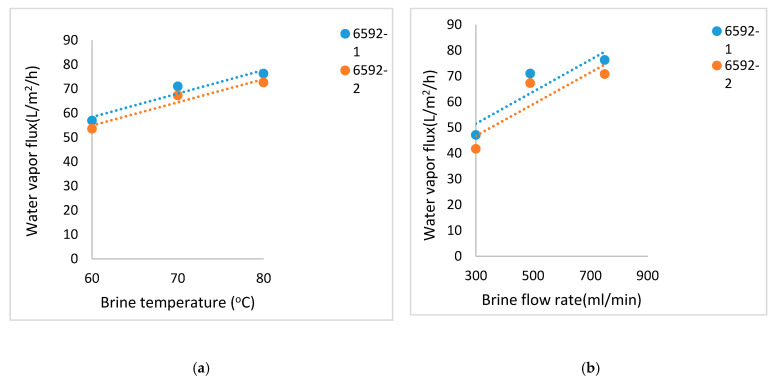
(**a**) Water vapor flux values for different brine temperatures and a constant brine flow rate of 490 mL/min for AKS- 6592-1 and AKS- 6592-2 Millipore. (**b**) Water vapor flux at various brine flow rates and a constant brine inlet temperature of 70 °C for AKS- 6592-1 and AKS- 6592-2 Millipore.

We show how the water vapor flux of the flat coated membranes varies with feed brine temperature and brine flow rate respectively in Figure 6a,b. The plasma polymerization levels for these membranes and therefore the coating thickness were just a bit higher than that of AKS-6591-1M and AKS-6591-2M membranes, which had the lowest plasma polymerization time, the lowest LEP value and the highest water vapor fluxes. We have already mentioned that the LEP values of these membranes (AKS-6591-1M and AKS-6591-2M) were comparable to those of the coated MicroPES HFMs of this study. Figure 6b shows how strongly the water vapor flux decreases at 70 °C with a decrease in feed brine flow rate and increase in temperature polarization. The cell design for flat membranes creates a significant amount of flow mixing to reduce temperature polarization. That is one of the reasons why the water vapor flux at 70 °C with the PES based hollow fiber modules in Figure 4 are smaller than those of flat membranes (Figure 6b).

**Figure 7:**
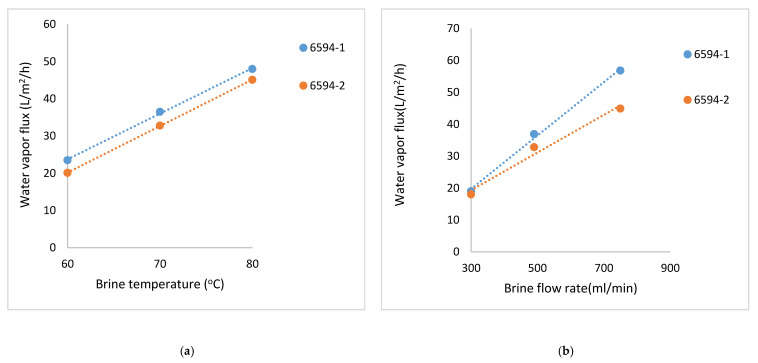
(**a**) Water vapor flux values for different brine temperatures and a constant brine flow rate of 490 mL/min for AKS- 6594-1 and AKS- 6594-2 Millipore. (**b**) Water vapor flux at various brine flow rates and a constant brine inlet temperature of 70 °C for AKS- 6594-1 and AKS- 6594-2 Millipore.

Figure 7a,b illustrate similar data for coated flat hydrophilic PVDF membranes designated AKS-6594-1 and AKS-6594-2 with Millipore substrate. The coatings were generated with a longer treatment time and hence led to lower fluxes. These coatings also show significantly higher LEP values [34]. In fact, one can now produce with reasonable certainty a certain type of plasma polymerized coating on a given substrate that will yield a certain small range of LEP and water vapor flux level, with due considerations for feed and distillate temperatures, and feed and distillate side fluid mechanical conditions.

## 4. Discussion

It is useful to deliberate on the volumetric distilled water production rate from the types of HFM-based modules used. Consider the distilled water flux from PES substrate-based modules in the 6900 series shown in Table 2. An average value of 27 kg/m^2^-h may be assumed from a module having a membrane surface area of 5.6 × 10^−3^ m^2^ and an internal volume of 15.32 cm^3^. The corresponding volumetric productivity is 9869 kg/m^3^-h. Considering that the volume fraction occupied by HFMs in these modules was only 5.17%, we can assume that this value can be easily tripled (or quadrupled) leading to a potential volumetric distilled water production rate of ~30,000 kg/m^3^-h. Such an estimate is useful for estimating the expected performance level from hollow fiber modules. It is considerably larger than modules based on flat membranes, spiral-wound or otherwise. Crossflow hollow fiber membrane modules designed and used earlier in DCMD can have a HFM packing fraction of ≥0.2 [35] in the cylindrical module. We recommend keeping the HFM packing volume fraction to ≤0.2 in view of considerations discussed in the next paragraph.

A related issue involves the pressure drop experienced by the hot brine flowing on the shell side of a HFM-based module. Since the hollow fiber bed has a relatively open cross-section, the pressure drop experienced by hot brine during passage through one module with a relatively deep fiber bed [13] is around ~13.6 kPa (2 psi). However, since the fractional water recovery in DCMD is not very high, a cascade of stages may be needed for high water recovery [19]. If at each stage, a pump is used to drive the brine, the pressure of the brine in any module will rarely exceed 10–15 kPag. Therefore, the LEP value of the current HFMs will not be exceeded. However, if reduced numbers of pumps are used, the hot brine pressure will be higher at the pump exit; that would require a higher value of LEP for coated MicroPES HFMs and a small reduction in achievable water vapor flux. These and other considerations on module design and system improvement of mem-brane distillation identified in [41] are of importance in further developments being undertaken.

The internal mechanism of water vapor flux in hydrophilic HFMs having a hydrophobic plasma polymerized coating in DCMD depends on water vapor diffusion through a thin gas-filled gap with colder water at the end of the thin gap. A transport model for DCMD in such a configuration has to take into account a number of items, which are unknown at this time: the thickness of the gas gap and the shape of its boundaries influenced by plasma coating; the thermal conductive resistance in parallel through the hydrophilic substrate membrane, as well as that through the water-filled pores of the substrate at the end of which distilled water flows. The temperature gradient in the water-filled pores of the substrate membrane is important as well since hot water vapor is condensing at the gas-liquid boundary inside the hydrophilic water-filled membrane pores while cold distilled water flows at the other end. Further, one has to be able to account for temperature polarization in the hot feed brine. This configuration thus requires an extensive investigation.

An aspect of plasma polymerization-based membrane surface modification is the general absence of the use of nanoparticles and organic solvents resulting in generation of toxic waste. Many advanced membranes being developed in MD involve production of such wastes. A recent example of MD membrane development that avoids/reduces such waste production is provided in [42].

## 5. Concluding Remarks

A very thin porous hydrophobic coating on a porous hydrophilic membrane can provide a membrane configuration for enhanced water vapor flux in DCMD with the hydrophilic substrate wetted by the cold distilled water stream. We demonstrated here that such a configuration can be achieved successfully with hydrophilic porous MicroPES hollow fiber membranes of polyethersulfone coated on the outside surface with a 0.4–0.5 µm thick porous hydrophobic polyfluorosiloxane coating using a vacuum-based plasma polymerization process. The HFM surface area in the module based on 20 HFMs was 5.6 × 10^−3^ m^2^. The water vapor flux level achieved with such hollow fiber membrane-based modules from a 1 wt% NaCl-containing brine feed at 70 °C was in the range of 26.4 to 27.6 kg/m^2^-h. These flux values were found to be significantly higher than those from hollow fiber membrane modules built using porous hydrophobic hollow fiber membranes of PP having a similar plasma polymerized coating; such PP-based hollow fibers require water vapor to traverse a long gas gap in the porous hydrophobic substrate. The water vapor fluxes from MicroPES hollow fibers are reduced to ~17–19 kg/m^2^-h when exposed to a 70 °C feed of 10 wt% of salt solution. The salt leakage was extremely low in both cases. The calculated values of the volumetric productivity of distilled water from such modules are substantial.

## Figures and Tables

**Table 1:** Properties of base hollow fiber membranes and flat membranes.

Base Membranes	d_m_ (µm) Mean Pore Size	ε_m_ (%) Porosity	δ_m_ (µm) Membrane Thickness	Internal Diameter (Hollow Fiber) (µm)
MicroPES hollow fiber (hydrophilic-polysulfone)	0.2	NA *	100 (wall thickness)	300
Polypropylene hollow fiber (hydrophobic)	>0.2	65	150 (wall thickness)	330
PVDF-MilliporeSigma (HVLP) (hydrophilic)	0.45	70	125	-
PVDF-Pall (hydrophilic)	0.1	82	72	-

* Not available.

**Table 2:** Average water vapor flux in DCMD for different polyethersulfone (PES)-based coated hollow fiber modules at two salt concentrations in feed at 70 °C.

Brine Concentration (%)	Module Series	Module Number	Type of Coating	Monomers Used	* W/FM	Flux (kg/m^2^-h)
1	6900	6935	Polysiloxane	TMDSO	0.024	27.6
		6939	Polyfluorosiloxane	TMCTS/HFE	0.018	26.4
		6940	Polysiloxane	TMDSO	0.030	26.7
10	6900	6924	Polysiloxane	TMDSO	0.037	16.5
		6932	Polyfluorosiloxane	TMDSO/HFE	0.025	15.3
		6933	Polyfluorosiloxane	TMDSO/HFE	0.019	19.5
		6935	Polysiloxane	TMDSO	0.024	17.0
		6939	Polyfluorosiloxane	TMDSO/HFE	0.018	17.1

* W/FM—W is the power used in watts; F is the monomer feed rate in SCCM; M is the molecular weight of the monomer or blend.

**Table 3:** Average water vapor flux in DCMD for different polypropylene (PP)-based coated hollow fiber modules at two feed salt concentrations at 70 °C.

Brine Concentration (%)	Module Series	Module Number	Type of Coating	Monomers Used	W/FM	Flux (kg/m^2^·h)
10	6700	6711	Polyfluorosiloxane	TMCTS/HFE	0.006	11.8
		6712	Polyfluorosiloxane	TMDSO/PFHX	0.008	10.1
		6713	Polyfluorosiloxane	TMDSO/PFHX	0.006	14.3
		6714	Polysiloxane	TMDSO	0.035	11.8
	6800	6823	Polyfluorosiloxane	TMDSO/PFHX	0.006	12.6
		6824	Polyfluorosiloxane	TMDSO/PFOC	0.006	11.4
		6825	Polyfluorosiloxane	TMDSO/PFOC	0.005	12.9
		6826	Polyfluorosiloxane	TMCTS */PFOC	0.006	15.5
20	6800	6803	Polyfluorosiloxane	TMDSO/PFHX	0.003	9.1
		6804	Polyfluorosiloxane	TMDSO/PFHX	0.007	7.6
		6805	Polyfluorosiloxane **	TMDSO/PFHX	0.007	10.1
		6806	Polyfluorosiloxane	TMCTS/PFHX	0.007	11.0
		6807	Polyfluorosiloxane **	TMCTS/PFHX	0.007	11.2
		6808	Polyfluorosiloxane	TMCTS/PFHX	0.007	10.1
		6810	Polyfluorosiloxane	TMDSO/PFHX	0.007	8.6

* Coating produced by reducing the amount of siloxane monomer by 50%; ** Reduced reaction time

**Table 4:** Data on liquid entry pressure (LEP) and leak rate in hollow fiber mini-modules * having selected coatings.

Hollow Fiber Membrane (HFM) Type	LEP Value in kPag (psig) for the Leak Rate Measured	Leak Rate, mL/h
Coated PES HFM, 6935	48.3 (7)	0.2
Coated PES HFM, 6939	48.3 (7)	1.1
Coated PES HFM, 6940	48.3 (7)	0.4
Uncoated PES HFM	48.3 (7)	4211
Coated PP HFM	68.9 (10)	0.2

* Each mini-module has 20 HFMs, 18 cm in length.

**Table 5:** Salt leak rate of polysiloxane and polyfluorosiloxane coating for PP based hollow fiber membrane at 70 °C.

Brine Concentration	Module/Coating No.	Coating Type	Average Water Flux (kg/m^2^·h)	Conductivity Change (4 h)
20%	6714	Polysiloxane	11.4	0.1–5.8 μs/cm
20%	6806	Polyfluorosiloxane	10.0	0.2–1.6 μs/cm

## References

[1] Yang X., Fane A.G., Wang R., Kucera J. (2018). Membrane Distillation: Now and Future. Desalination.

[2] Drioli E., Ali A.A.A., Macedonio F. (2015). Membrane distillation: Recent developments and perspectives. Desalination.

[3] Deshmukh A., Boo C., Karanikola V., Lin S., Straub A.P., Tong T., Warsinger D.M., Elimelech M. (2018). Membrane distillation at the water-energy nexus: Limits, opportunities, and challenges. Energy Environ. Sci..

[4] Camacho L.M., Dumée L.F., Zhang J., Li J.-D., Duke M., Gomez J.D., Gray S. (2013). Advances in Membrane Distillation for Water Desalination and Purification Applications. Water.

[5] Alkhudhiri A., Darwish N.A., Hilal N. (2012). Membrane distillation: A comprehensive review. Desalination.

[6] Lawson K.W., Lloyd D.R. (1997). Membrane distillation. J. Membr. Sci..

[7] Sirkar K.K., Ho W.S.W., Sirkar K.K. (2012). Other New Membrane Processes. Membrane Handbook.

[8] Chen H.-C., Chen Y.-R., Yang K.-H., Yang C.-P., Tung K.-L., Lee M.-J., Shih J.-H., Liu Y.-C. (2018). Effective reduction of water molecules’ interaction for efficient water evaporation in desalination. Desalination.

[9] Anvari A., Kekre K.M., Yancheshme A.A., Yao Y., Ronen A. (2019). Membrane distillation of high salinity water by induction heated thermally conducting membranes. J. Membr. Sci..

[10] Dudchenko A.V., Chen C., Cardenas A., Rolf J., Jassby D. (2017). Frequency-dependent stability of CNT Joule heaters in ionizable media and desalination processes. Nat. Nanotechnol..

[11] Singh D., Prakash P., Sirkar K.K. (2013). De-oiled Produced Water Treatment using Direct Contact Membrane Distillation. I&EC Res..

[12] He F., Gilron J., Lee H., Song L., Sirkar K. (2008). Potential for scaling by sparingly soluble salts in crossflow DCMD. J. Membr. Sci..

[13] Song L., Ma Z., Liao X., Kosaraju P.B., Irish J.R., Sirkar K.K. (2008). Pilot plant studies of novel membranes and devices for direct contact membrane distillation-based desalination. J. Membr. Sci..

[14] He F., Sirkar K., Gilron J. (2009). Effects of antiscalants to mitigate membrane scaling by direct contact membrane distillation. J. Membr. Sci..

[15] Wang Z., Chen Y., Sun X., Duddu R., Lin S. (2018). Mechanism of pore wetting in membrane distillation with alcohol vs. surfactant. J. Membr. Sci..

[16] Chen Y., Tian M., Li X., Wang Y., An A.K., Fang J., He T. (2017). Anti-wetting behavior of negatively charged superhydrophobic PVDF membranes in direct contact membrane distillation of emulsified wastewaters. J. Membr. Sci..

[17] Mostafa M.G., Zhu B., Cran M.J., Dow N., Milne N.A., Desai D., Duke M. (2017). Membrane Distillation of Meat Industry Effluent with Hydrophilic Polyurethane Coated Polytetrafluoroethylene Membranes. Membranes.

[18] Tang L., Iddya A., Zhu X., Dudchenko A.V., Duan W., Turchi C., Vanneste J., Cath T.Y., Jassby D. (2017). Enhanced Flux and Electrochemical Cleaning of Silicate Scaling on Carbon Nanotube-Coated Membrane Distillation Membranes Treating Geothermal Brines. ACS Appl. Mater. Interfaces.

[19] He F., Gilron J., Sirkar K.K. (2013). High water recovery in direct contact membrane distillation using a series of cascades. Desalination.

[20] Winter D., Koschikowski J., Gross F., Maucher D., Düver D., Jositz M., Mann T., Hagedorn A. (2017). Comparative analysis of full-scale membrane distillation contactors—methods and modules. J. Membr. Sci..

[21] Singh D., Sirkar K.K. (2012). Desalination by Airgap Membrane Distillation Using a Two Hollow-fiber-set Membrane Module. J. Membr. Sci..

[22] Schwantes R., Seger J., Bauer L., Winter D., Hogen T., Koschikowski J., Geißen S.-U. (2019). Characterization and Assessment of a Novel Plate and Frame MD Module for Single Pass Wastewater Concentration–FEED Gap Air Gap Membrane Distillation. Membranes.

[23] Li L., Sirkar K.K. (2016). Influence of microporous membrane properties on the desalination performance in direct contact membrane distillation. J. Membr. Sci..

[24] Lee H., He F., Song L., Gilron J., Sirkar K. (2010). Desalination with a cascade of cross-flow hollow fiber membrane distillation devices integrated with a heat exchanger. AIChE J..

[25] Khayet M., Mengual J., Matsuura T. (2005). Porous Hydrophobic/hydrophilic Composite Membranes- Application in Desalination using Direct Contact Membrane Distillation. J. Membr. Sci..

[26] Khayet M., Matsuura T., Mengual J., Qtaishat M. (2006). Design of novel direct contact membrane distillation membranes. Desalination.

[27] Khayet M., Matsuura T., Qtaishat M.R., Mengual J.I. (2006). Porous hydrophobic/hydrophilic composite membranes preparation and application in DCMD desalination at higher temperatures. Desalination.

[28] Qtaishat M., Khayet M., Matsuura T. (2009). Guidelines for preparation of higher flux hydrophobic/hydrophilic composite membranes for membrane distillation. J. Membr. Sci..

[29] Floros I.N., Kouvelos E.P., Pilatos G.I., Hadjigeorgiou E.P., Gotzias A.D., Favvas E.P., Sapalidis A.A. (2020). Enhancement of Flux Performance in PTFE Membranes for Direct Contact Membrane Distillation. Polymers.

[30] Liu Y., Xiao T., Bao C., Fu Y., Yang X. (2018). Fabrication of novel Janus membrane by nonsolvent thermally induced phase separation (NTIPS) for enhanced performance in membrane distillation. J. Membr. Sci..

[31] Tian M., Yin Y., Yang C., Zhao B., Song J., Liu J., Li X.-M., He T. (2015). CF4 plasma modified highly interconnective porous polysulfone membranes for direct contact membrane distillation (DCMD). Desalination.

[32] Eykens L., De Sitter K., Dotremont C., Pinoy L., Van Der Bruggen B. (2018). Coating techniques for membrane distillation: An experimental assessment. Sep. Purif. Technol..

[33] Wei X., Zhao B., Li X.-M., Wang Z., He B.-Q., He T., Jiang B. (2012). CF4 plasma surface modification of asymmetric hydrophilic polyethersulfone membranes for direct contact membrane distillation. J. Membr. Sci..

[34] Puranik A.A., Rodrigues L.N., Chau J., Li L., Sirkar K.K. (2019). Porous hydrophobic-hydrophilic composite membranes for direct contact membrane distillation. J. Membr. Sci..

[35] Singh D., Li L., Obusckovic G., Chau J., Sirkar K.K. (2018). Novel cylindrical cross-flow hollow fiber membrane module for direct contact membrane distillation-based desalination. J. Membr. Sci..

[36] Sharma A.K., Yasuda H. (1989). Polymerization of methane. J. Appl. Polym. Sci..

[37] Sharma A.K., Yasuda H. (1983). Plasma polymerization of tetramethyldisiloxane by a magnetron glow discharge. Thin Solid Films.

[38] Sharma A.K., Millich F., Hellmuth E.W. (1979). Propylene Glow Discharge Polymerization in the Presence of Bromotrichloromethane. ACS Symp. Ser..

[39] Sharma A.K. (2016). Organosiloxane Films for Gas Separations. U.S. Patent.

[40] Sharma A.K., Millich F., Hellmuth E.W. (1978). Adhesion and Hydrophobicity of Glow Discharge Polymerized Propylene Coating. J. Appl. Phys..

[41] Lu K.J., Chung T.S. (2020). Membrane Distillation: Membranes, Hybrid Systems and Pilot Studies.

[42] Lu K., Zhao D., Chen Y., Chang J., Chung T.-S. (2020). Rheologically controlled design of nature-inspired superhydrophobic and self-cleaning membranes for clean water production. npj Clean Water.

